# Isolation and identification of Pasteurella species and other bacterial organisms from ovine pneumonic lungs and their antimicrobial susceptibility in Ghana: abattoir-based study

**DOI:** 10.3389/fvets.2026.1834690

**Published:** 2026-05-08

**Authors:** Abass Abdulai, Benjamin Obukowho Emikpe, Raphael Deladem Folitse

**Affiliations:** 1 School of Veterinary Sciences, University for Development Studies, Tamale, Ghana; 2 School of Veterinary Medicine, Kwame Nkrumah University of Science and Technology, Kumasi, Ghana

**Keywords:** antibiotic, bacteria, isolation, ovine, pneumonia, susceptibility

## Abstract

**Background and aim:**

Pneumonia is the most prevalent respiratory infection in sheep, significantly impacting their health and welfare. This study aimed to isolate Pasteurella spp. and other bacterial organisms from pneumonic sheep lungs and also assess their antibiotic susceptibility.

**Materials and methods:**

A cross-sectional abattoir-based study was conducted at Tamale and Kumasi abattoirs. Seventy-five ovine pneumonic lungs were randomly sampled to culture and isolate bacterial organisms associated with ovine pneumonia. The Kirby-Bauer disk diffusion method was utilized to evaluate antibiotic susceptibility.

**Results:**

Among the 75 pneumonic lungs that were cultured, *Mannheimia haemolytica* was the predominant Pasteurella species, isolated from 54.7% of samples. Other bacteria identified included *Staphylococcus aureus* (46.7%), *E. coli* (37.3%), and *Klebsiella spp*. (19.7%). *M. haemolytica* was particularly associated with broncho-interstitial pneumonia (29.3%) and interstitial pneumonia (17.3%), while *P. multocida* was prominent in interstitial cases (13.3%). Both *M. haemolytica* and *P. multocida* contributed to 8.0% of bronchopneumonia cases. Seasonal patterns showed *M. haemolytica* (30%) and *P. multocida* (16%) were more common in the dry season, whereas *Staphylococcus aureus* was more prevalent in the wet season. *M. haemolytica and P. multocida* were most frequently identified in 2–3-year-old sheep from Sahelian breeds. All bacteria were isolated from sheep in poor body condition, but no significant associations were found between breeds, body condition, and pneumonia forms (*p* > 0.05). *M. haemolytica* and *P. multocida* were highly susceptible to cefotaxime (95%, 100%) and ciprofloxacin (83%, 91%), but exhibited resistance to amoxicillin and doxycycline. *Staphylococcus aureus* showed sensitivity to gentamicin (94%) and cefoxitin (71%), but resistance to tetracycline (63%). *Pseudomonas spp*. was completely susceptible to gentamicin, meropenem, and amikacin (all 100%) while resistant to tetracycline (100%) and sulfamethoxazole (75%).

**Conclusion:**

*Mannheimia haemolytica* was the most prevalent bacterial pathogen isolated from pneumonic sheep lungs in this study, suggesting its primary role in ovine pneumonia. *Staphylococcus aureus, E. coli, Klebsiella spp., Pseudomonas spp., Enterobacter*, and *Citrobacter spp* are also prevalent. These bacteria are mostly effectively inhibited by antibiotics such as cefotaxime, ciprofloxacin, and gentamicin, but they are not susceptible to amoxicillin, tetracycline, and penicillin G.

## 1 Introduction

Pneumonia is a respiratory infection that adversely affects the health, welfare, and production of sheep. This situation is exacerbated in developing countries, where veterinary services are limited and small ruminants are raised for income and food security (1). Sheep are predisposed to pneumonia due to factors like transportation, overcrowding, temperature fluctuations, starvation, and poor ventilation. Infectious agents can act as primary pathogens or as opportunistic invaders, particularly when the host's upper defenses are down (2). Viruses and mycoplasmas damage the respiratory system, which enables bacteria to settle. Acute fibrinous pneumonia is primarily caused by *Mannheimia haemolytica*, whereas chronic suppurative pneumonia is associated with *Pasteurella multocida* (3). Other bacteria involved with sheep respiratory diseases include *Bibersteinia trehalosi* and *Streptococcus spp*. (4) and are usually detected in slaughterhouse surveillance.

In sheep-rearing countries, including Ghana, pneumonia leads to economic losses due to the death of sheep, lower carcass weight, poor growth rates, consolidation of the lungs that must be condemned at slaughter, and high treatment costs (5). However, there is limited information regarding specific causes of ovine pneumonia in Ghana. Identifying bacterial organisms associated with pneumonia is vital for accurate diagnosis, treatment and control of the disease. In addition, the rising reports of antimicrobial resistance in both animal and human bacteria in Ghana emphasize the need for evidence-based antibiotic selection in veterinary practice. Therefore, determining a specific bacterial organism associated with pneumonic lungs in sheep and evaluating their resistance patterns is essential. This information will guide effective treatment, reduce losses in sheep production and support national surveillance efforts on antimicrobial resistance. This study enhances the understanding of ovine pneumonia and its related antimicrobial resistance (AMR) in Ghana by presenting context-specific data derived from sheep lungs obtained from abattoirs in Tamale and Kumasi. It establishes a direct connection between pathological lesions and bacterial pathogens along with their AMR profiles, following CLSI veterinary standards. In contrast to earlier studies that mainly provide general prevalence information on AMR across human, animal, and environmental domains in Ghana without integrating pathology, or that concentrate on pneumonia pathology in specific regions without AMR details, our results highlight resistance patterns that are specific to lesions, such as those found in *Pasteurella spp*. and other bacterial organisms. This focus on the relationship between pathology and AMR in Ghana addresses a significant gap in the veterinary literature of West Africa, facilitating targeted interventions that go beyond broad prevalence statistics.

The study, therefore, aimed to identify *Pasteurella spp*. and other bacterial organisms associated with pneumonia in sheep slaughtered at abattoirs and their antimicrobial susceptibility in Ghana.

## 2 Materials and methods

### 2.1 Ethical approval

Ethical approval was not sought because the samples were collected from lungs condemned during routine abattoir meat inspection, and no procedures were performed on live animals. During meat inspection, lungs with consolidated areas were completely condemned, and these condemned tissues were then collected for histopathological and microbiological examination. However, samples were collected aseptically in accordance with established collection methods.

### 2.2 Study areas

Tamale Abattoir and Kumasi Abattoir Company Limited (KACL) were the study locations. The Tamale metropolis lies between latitudes 9 °0.15′ and 9 °0.30′N and between longitudes 0 °0.45′ and 10 °W, at an altitude of 183.3 m above sea level. The area is characterized by guinea savannah with two main seasons: erratic rain from April to October, with 1,000 mm of rainfall and a short dry season from November to March (6). The Tamale abattoir facility has slaughtering sections for large and small ruminants, which operate every day.

Kumasi Abattoir Company Limited (KACL) is another important facility established in 1997 with the assistance of the Government of Ghana and the Canadian International Development Agency (CIDA). The company began operations in 1998 in the Ahinsan/Kaase Industrial Area, which is situated at 6 °9′36.6 °N latitude and 1 °6′15.4 °W longitude in Kumasis, the Ashanti region of Ghana. It is among the largest and busiest abattoirs in the country (7). The selected study locations have varied climatic conditions, vegetative cover and environmental conditions and can give representative national data. However, because this was an abattoir-based study using purposive sampling of lungs with gross pneumonic lesions, the findings are representative of sheep presented for slaughter with pneumonia at these abattoirs rather than of the wider live sheep population in Ghana.

### 2.3 Study design

A cross-sectional abattoir-based study was conducted for this investigation's purposes. The study was conducted from May 2023 to April 2024. All sheep slaughtered during the study period were considered. However, sheep slaughtered with lung consolidations were systematically sampled for histopathological and bacteriological examination.

#### 2.3.1 Inclusion and exclusion criteria for lung selection

During routine meat inspection, any sheep lung showing consolidation in one or more lobes, or affecting the entire lung, was included in the study, whereas lungs without visible consolidation were excluded.

### 2.4 Study animal and sampling technique

A total of 3,038 lungs (an average of 9 sheep slaughtered per day) from sheep of different breeds, ages, and sexes were examined at the abattoirs during the meat inspection conducted by veterinary professionals. The purposive sampling technique was used to identify 251 ovine lungs with consolidations. Grossly, a total of 65 out of 251 consolidated lungs exhibited cranioventral lobe consolidation, ranging in color from red to gray. These lungs were difficult to cut due to thickened interlobular septa and pleura. Additionally, 84 of the consolidated lungs had a rubbery consistency and a meaty appearance. Furthermore, 102 consolidated lungs appeared meaty, remained uncollapsed, and showed consolidation toward the anterior of the cranial lobes. These consolidated lungs were then subjected to histopathological examination to identify pneumonia histotypes.

A subset of 75 out of the 251 pneumonic lungs was selected for microbiological examination. To ensure this subset was representative of the different histopathological forms of pneumonia, we used stratified random sampling by histotype. First, all pneumonic lungs were classified as bronchopneumonia, interstitial pneumonia, or broncho-interstitial pneumonia. Within each histotype stratum, lung identification numbers were then randomly selected using the R software random number generator to obtain a total of 75 samples, with an approximate proportional allocation of 30% bronchopneumonia, 30% interstitial, and 40% broncho-interstitial lesions. This microbiological component was designed as an exploratory study to provide baseline information on the bacterial species associated with ovine pneumonia and their antimicrobial susceptibility patterns. The microbiological examination was limited to 75 lung samples from pneumonia cases due to resource constraints, including the high costs of specialized media and selective broths.

### 2.5 Study duration and data collection

Meat inspection was conducted daily on sheep slaughtered at the abattoirs for a year. The study year was divided into two (2) seasons, with data collected from May to October, for the wet season and from November to April for the dry season.

Meat inspection was performed by the veterinary and para-veterinary professionals at each abattoir, and they assisted in identifying consolidated lungs. Details about sheep slaughtered with lung consolidation were recorded, including age, sex, breed, season, and body condition score. The age was determined by using their dentition. Breed differences between the West African Dwarf sheep and the Sahelian and their crosses were determined by examining the differences in the length of the fore and hind legs of sheep before and after slaughter. The amount of fat and muscle around the loin, as well as the general state of the body, was assessed and scored on a scale of 1–4: 1 = very thin (bad), 2 = thin (fair), 3 = normal (good), and 4 = fat (obese) (8).

### 2.6 Sample collection for histopathology

At the abattoirs, each consolidated lung from a sheep was divided into two samples, one section for histopathology and the other section for microbiological examination. For histopathological examination, a section was stored in labeled containers with 10% buffered formalin. The samples were taken to the histopathology laboratory at the School of Veterinary Medicine, Kwame Nkrumah University of Science and Technology (KNUST) for processing.

#### 2.6.1 Histopathology examination

Lung samples collected in 10% formalin were examined histopathologically (8). After grossing, samples were fixed in neutral buffered formalin for 24 h, followed by dehydration in graded alcohol concentrations (70%, 80%, 90%, 95%, 95%, and 100%) for 1 h each. The tissues were then cleared in xylene for 2 h before being infiltrated with molten paraffin wax in a wax oven for 4 h. After embedding, the tissues were sectioned at 5 μm using a microtome (Leica) and floated in a water bath before being mounted on frosted-edge slides and labeled. The slides were dewaxed in xylene (twice) for 15 min and rehydrated in a decreasing gradient of ethanol (100%, 100%, 80%, 70%). The samples were then stained with hematoxylin, differentiated in 1% acid alcohol, counterstained with 1% eosin and dehydrated in ethanol before mounting with DPX mounting medium. Pathology was visualized under an Olympus CX2 microscope, photographed with an Amscope MU900. Pulmonary lesions of each sheep were described, categorized and diagnosed according to their morphology.

### 2.7 Pathology

Consolidated lung samples were initially identified at the abattoirs and then confirmed through microscopic histopathological examination. Various histotypes, including bronchopneumonia, interstitial pneumonia, and broncho-interstitial pneumonia, were identified. The pneumonic lung samples were also collected with consideration to factors such as season, sex, age, breed, and body condition score.

For microbiological examination, one section of the consolidated lung samples was placed in a labeled zip-lock bag and stored in an ice chest containing ice blocks for transport to the Kumasi Veterinary Laboratory. Upon arrival at the laboratory, the samples were stored at −20 °C to maintain their integrity for bacteriological examination. The pneumonic status of the consolidated lungs selected for microbiological examination was revealed after the histopathological examination.

### 2.8 Bacteriological examination and isolation of bacteria

The isolation and differentiation of bacterial organisms were carried out according to (9, 10). Pneumonic lung tissue samples were aseptically extracted at 37 °C for 24 h to serve as an enrichment step using a sterile scalpel and forceps, then transferred to a sterile test tube with 5 mL of tryptose broth. A loop of the broth was then inoculated onto nutrient agar and blood agar, followed by aerobic incubation at 37 °C for 24 h. All morphologically distinct colonies obtained from each pneumonic lung sample were further identified, and a single lung could therefore yield one or more bacterial species. The colonies were subcultured onto blood agar enriched with 7% sheep blood, MacConkey agar, and Mannitol salt agar, and incubated at 37 °C for 24 to 48 h. Characteristics of the organism on MacConkey agar were subcultured on brilliant UTI agar for presumptive identification of *Staphylococcus spp., Klebsiella spp.*, and *Pseudomonas spp* from enrichment broth cultures of a pneumonic lung sample. ECC agar, which is chromogenic agar, was also utilized to distinguish *E. coli* from other organisms. MIU and oxidase tests were used as a secondary confirmation of the chrome agar identification. For the other members of the Enterobacteriaceae, which are all gram-negative bacteria, an oxidase test confirms their presence and MU, citrate, TSI and MR tests were used to confirm their identity based on the reaction to these biochemical assays.

For *Staphylococcus aureus*, all presumptive isolates appeared as Gram-positive cocci in clusters and were catalase positive with rapid bubble formation in 3% hydrogen peroxide. They also demonstrate urease positivity with a pink color change in urea medium. *Pseudomonas spp*. was identified based on their characteristic colonies and pigment production on Mueller-Hinton agar together with oxidase, catalase, TSI, citrate and motility tests.

Quality control for bacteriological identification was ensured by testing standard reference strains such as E. coli ATCC 25922 and Staphylococcus aureus ATCC 25923, together with field isolates. These strains were used to verify the effectiveness of culture media and biochemical tests, in line with laboratory guidelines.

#### 2.8.1 Isolation and Identification of *Pasteurella spp*

The colonies on blood agar and MacConkey agar were examined for hemolysis, morphology, color, shape, size, consistency, and other growth characteristics. Presumptive *Mannheimia haemolytica* colonies were identified based on characteristic morphology on blood and MacConkey agar. On 5% ovine blood agar, isolates appeared as small (1–2 mm), gray, round colonies surrounded by a clear zone of complete β-hemolysis after 24–48 h incubation at 37 °C. On MacConkey agar, they produced small, pink, pinpoint-to-slightly mucoid colonies, consistent with weak lactose fermentation and comparatively poorer growth than that of Enterobacteriaceae. Presumptive *Pasteurella multocida* colonies were identified based on their failure to grow on MacConkey agar and their characteristic appearance on blood agar. On 5% ovine blood agar, isolates appeared as round, smooth, grayish, non-hemolytic colonies, 1–3 mm in diameter after 24–48 h incubation at 37 °C, often with a glistening or slightly mucoid surface and a ‘mousy' odor. No colony growth was observed on MacConkey agar, which helped differentiate *P. multocida* from *M. haemolytica* and enteric Gram-negative organisms in mixed cultures.

Finally, pure cultures grown on nutrient agar were subjected to confirmatory biochemical assays, such as catalase and oxidase tests, culminating in the Indole production test for definitive species identification: its absence confirms the isolate as *M. haemolytica*, whereas its presence confirms it as *P. multocida* (11). To achieve species-level identification, we relied on this combination of conventional phenotypic criteria (colony morphology, haemolysis, growth on MacConkey agar, and basic biochemical reactions), which are routinely used in veterinary diagnostic laboratories, while acknowledging that these methods are less specific than molecular techniques such as 16S rRNA gene sequencing.

### 2.9 Antimicrobial susceptibility of bacterial organisms

Antimicrobial susceptibility testing was performed using commercially available antibiotic disks (Oxoid Ltd., Basingstoke, UK), supplied in sealed cartridges with desiccant. Disks were transported and stored according to the manufacturer's instructions, in a sealed, dry container at 2–8 °C, and equilibrated to room temperature before use. Only disks within their stated expiry date were used for testing. Antimicrobial susceptibility testing was performed using the Kirby–Bauer disk diffusion method in accordance with CLSI veterinary standards (12). For *Mannheimia haemolytica* and *Pasteurella multocida*, testing was conducted on Mueller–Hinton agar supplemented with 5% defibrinated sheep blood, with inocula adjusted to a 0.5 McFarland standard and incubation at 35–37 °C for 18–24 h, and zone diameters interpreted according to CLSI VET01/VET01S recommendations for fastidious respiratory pathogens isolated from animals (13). For *Staphylococcus aureus, E. coli, Klebsiella spp., Pseudomonas spp*. and other non-fastidious isolates, AST followed CLSI VET01 using standard Mueller–Hinton agar and interpretive breakpoints from the current CLSI veterinary performance tables (VET01S) for *Staphylococcus spp*., Enterobacteriaceae, and *Pseudomonas spp* (12).

Each isolate was tested against antibiotic disks commonly used to treat pneumonia. For *Pasteurella spp.*, eight different antibiotics were tested, including ampicillin (30 μg), amoxicillin (10 μg), cefotaxime (5 μg), and penicillin G (10 IU), among others. We applied CLSI VET01/VET01S Pasteurellaceae breakpoints for Mannheimia haemolytica and Pasteurella multocida wherever they were available, and for organism–antibiotic–disc content combinations lacking a CLSI veterinary disk-diffusion breakpoint (e.g., amoxicillin 30 μg), we reported only the measured zone diameters without assigning CLSI susceptible/resistance categories. *Staphylococcus aureus* and *Pseudomonas spp*. were tested against seven antibiotics, which included ciprofloxacin (5 μg), tetracycline (10 μg), gentamicin (10 μg), and sulfamethoxazole (25 μg), among others. Additionally, *Klebsiella spp*., *E. coli*, and *Citrobacter spp*. were tested against seven antibiotics, including ciprofloxacin (5 μg), tetracycline (30 μg), gentamicin (10 μg), and ceftriaxone (30 μg), among others.

### 2.10 Data analysis

The data was analyzed using SPSS (IBM, version 22). Descriptive analysis was conducted to estimate the percentages and frequencies by using cross-tabulation. The number and percentages of the bacterial isolates were presented in frequencies and percentages. Chi-square of Pearson (X^2^) test (test of association) was used to compare the rates of bacterial isolates in different pneumonic histotypes, seasons, ages, breeds, and sexes as predisposing factors at α = 0.05. The data were categorized based on the pathogens according to their susceptibility patterns for each class of antibiotics, which were classified as resistant, susceptible, or intermediate.

## 3 Results

### 3.1 Histopathological examination

Histopathological examinations were conducted on the lungs of 251 sheep that had been consolidated. The most common histotype was broncho-interstitial pneumonia (40.6%), which was followed by interstitial pneumonia (33.5%) and bronchopneumonia (25.9%). Multinucleated syncytial cells within alveolar gaps, neutrophils and fibrin in alveolar lumina and airways, and lymphocytes and macrophages thickening alveolar walls were the characteristics of broncho-interstitial lesions. While bronchopneumonia was characterized by neutrophil and macrophage-rich exudates in bronchiolar lumina and alveolar spaces with enlarged, inflammatory interlobular septa, interstitial pneumonia was associated with enlargement of interalveolar septa by mostly lymphocytes and macrophages with fewer neutrophils. These histopathological patterns in the same population have been described in more detail previously (14).

### 3.2 Bacterial organism isolated

A total of eight (8) different bacterial organisms were isolated from the pneumonic lung samples. *Mannheimia haemolytica* (54.7%) and *P. multocida* (30.7%) were the *Pasteurella spp*. associated with pneumonia. Other bacterial organisms identified included *Staphylococcus aureus* (46.7%), *E. coli* (37.3%), and *Klebsiella spp*. (19.7%). Because individual lungs often yielded more than one bacterial species, the percentages for each organism represent the proportion of the 75 lungs in which that organism was detected and therefore do not sum to 100%. These findings are shown in Table 1.

**Table 1:** Bacterial organisms isolated from ovine pneumonic lungs.

Bacterial organism	Number of positives	Percentage (%)
*Mannheimia haemolytica*	41	54.7
*Pasteurella multocida*	23	30.7
*Staphylococcus aureus*	35	46.7
*Escherichia coli*	28	37.3
*Klebsiella spp*	15	19.7
*Citrobacter spp*	7	9.3
*Pseudomonas spp*	4	5.3
*Enterobacter spp*	3	4.0

The total number of samples examined was 75.

### 3.3 The relationships between bacterial organisms in different histotypes of ovine pneumonia and the associated risk factors

*Manheimia haemolytica* was the main organism associated with broncho-interstitial pneumonia (29.3%) and interstitial pneumonia (13.3%). *P. multocida* also played a role in interstitial pneumonia (13.3%) and broncho-interstitial pneumonia (9.3%). Both *M. haemolytica* and *P. multocida* contributed to bronchopneumonia (8.0% each). *Staphylococcus aureus* and *E. coli* were present in all pneumonia types, while *Klebsiella spp*. and *Pseudomonas spp*. were absent in broncho-interstitial pneumonia. There was no significant association between bacterial organisms and pneumonia forms, *p* > 0.05 (Table 2).

**Table 2:** Association between the bacterial organism and histotypes with the frequency and percentage of negative and positive samples.

Bacterial isolates	Status	Histotypes			*X* ^2^	*P*-value
		Broncho interstitial freq (%)	Bronchopneumonia freq (%)	Interstitial pneumonia freq (%)		
*M. haemlytica*	Absent	12 (16.0)	10 (13.3)	12 (16.0)	3.357	0.187
	Present	22 (29.3)	6 (8.0)	13 (17.3)		
*P. multocida*	Absent	27 (36.0)	10 (13.3)	15 (20.0)	3.000	0.223
	Present	7 (9.3)	6 (8.0)	10 (13.3)		
*S. aureus*	Absent	18 (24.0)	5 (6.7)	17 (22.7)	5.298	0.071
	Present	16 (21.3)	11 (14.7)	8 (10.7)		
*E. coli*	Absent	21 (28.0)	10 (13.3)	16 (21.3)	0.031	0.985
	Present	13 (17.3)	6 (8.0)	9 (12.0)		
*Klebsiella spp*.	Absent	0 (0)	30 (39.5)	12 (15.8)	2.727	0.600
	Present	0 (0.0)	4 (5.3)	4 (5.3)		
*Enterobacter. Spp*	Absent	32 (42.7)	15 (20.0)	25 (33.3)	1.566	0.457
	Present	3 (4.0)	2 (2.7)	2 (2.7)		
*Pseudomonas. Spp*.	Absent	34 (45.3)	15 (20.0)	22 (29.3)	4.143	0.126
	Present	0 (0.0)	1 (1.3)	3 (4.0)		
*Citrobacter spp*	Absent	31 (41.3)	14 (18.7)	23 (30.7)	1.566	0.457
	Present	2 (2.7)	1 (1.3)	0 (0.0)		

No (frequency).

In Table 3, *M. haemolytica* (30%) and *P. multocida* (16%) were primarily found during the dry season, while Staphylococcus aureus (46.7%) was dominant in the wet season. *Klebsiella spp*. (13.3%) was also more common in the dry season, and *Pseudomonas spp*. was absent in the wet season.

**Table 3:** Associations between bacterial organisms and the season.

Bacterial isolates	Status	Season		*X* ^2^	*P*-value
	Dry freq (%)	Wet freq (%)		
*M. haemolytica*	Absent	18 (24.0)	16 (21.3)	0.075	0.484
	Present	23 (30.7)	18 (24.0)		
*P. multocida*	Absent	29 (38.7)	23 (30.7)	0.083	0.484
	Present	12 (16.0)	11 (14.7)		
*S. aureus*	Absent	23 (30.7)	17 (22.7)	0.278	0.384
	Present	18 (24.0)	25 (33.3)		
*E. coli*	Absent	27 (36.0)	20 (26.7)	0.393	0.349
	Present	14 (18.7)	14 (18.7)		
*Klebsiella spp*.	Absent	31 (41.3)	29 (38.7)	1.089	0.227
	Present	10 (13.3)	5 (6.7)		
*Enterobacter spp*.	Absent	39 (52.0)	33 (44.0)	0.018	0.571
	Present	4 (5.3)	3 (4.0)		
*Pseudomonas spp*.	Absent	37 (49.3)	34 (45.3)	3.504	0.083
	Present	4 (5.3)	0 (0.0)		
*Citrobacter spp*.	Absent	37 (49.3)	31 (41.3)	0.019	0.605
	Present	2 (2.7)	1 (1.3)		

*M. haemolytica* was the most frequently identified among all breeds, with all bacteria isolated from Sahelian breeds, except for *Enterobacter spp*. as shown in Table 4.

**Table 4:** Associations between bacterial organisms and the sheep breeds.

Bacterial isolates	Status	Breeds			*X* ^2^	*P*-value
	Wds freq (%)	Sahelian freq (%)	Cross freq (%)		
*M. haemolytica*	Absent	20 (26.7)	8 (10.7)	6 (8.0)	0.644	0.725
	Present	21 (28.0)	13 (17.3)	7 (9.3)		
*P. multocida*	Absent	27 (36.0)	16 (21.3)	9 (12.0)	0.698	0.705
	Present	14 (18.7)	5 (6.7)	4 (5.3)		
*S. aureus*	Absent	22 (29.3)	11 (14.7)	7 (9.3)	0.011	0.995
	Present	19 (25.3)	10 (13.3)	6 (8.0)		
*E. coli*	Absent	23 (30.7)	14 (18.7)	10 (21.3)	2.029	0.363
	Present	18 (24.0)	7 (9.3)	3 (4.0)		
*Klebsiella spp*.	Absent	35 (46.1)	16 (21.1)	9 (11.8)	77.896	<0.001
	Present	6 (7.9)	5 (6.6)	4 (5.3)		
*Enterobacter spp*.	Absent	39 (52.0)	20 (26.7)	13 (17.3)	0.656	0.720
	Present	0 (0.0)	1 (1.3)	2 (2.7)		
*Pseudomonas spp*	Absent	38 (50.7)	20 (26.7)	13 (17.3)	1.066	0.587
	Present	3 (4.0)	1 (1.33)	0 (0.0)		
*Citrobacter spp*	Absent	38 (50.7)	19 (25.3)	11 (14.7)	0.760	0.684
	Present	2 (2.7)	2 (2.7)	3 (4.0)		

Table 5 shows that *M. haemolytica, P. multocida, Staphylococcus aureus*, and *E. coli* were the most common pathogens in 2–3-year-old sheep. *P. multocida* was absent in sheep older than 4 years. *Klebsiella spp*. was significantly associated with age, *p* > 0.05. Most bacterial organisms were found in female sheep, except for *Citrobacter spp* (Table 6).

**Table 5:** Associations between bacterial organisms and the sheep age.

Bacterial isolates	Status	Age (years)					X^2^	*P*-value
	0–1 freq (%)	1–2 freq (%)	2–3 freq (%)	3–4 freq (%)	>4 freq (%)		
*M. haemolytica*	Absent	6 (8.0)	11 (14.7)	10 (13.3)	5 (6.7)	2 (2.7)	4.658	0.324
	Present	5 (6.7)	12 (16.0)	12 (16.0)	3 (4.0)	9 (12.0)		
*P. multocida*	Absent	7 (9.3)	17 (22.7)	13 (17.3)	4 (5.3)	11 (14.7)	7.752	0.101
	Present	4 (5.3)	6 (8.0)	9 (12.0)	4 (5.3)	0 (0)		
*S. aureus*	Absent	7 (9.3)	12 (16.0)	12 (16.)	4 (5.3)	5 (6.7)	0.805	0.938
	Present	4 (14.7)	23 (30.7)	22 (29.3)	8 (10.7)	6 (8.0)		
*E. coli*	Absent	8 (10.7)	15 (20.0)	10 (13.3)	6 (8.0)	8 (10.7)	4.322	0.364
	Present	3 (4.0)	8 (10.7)	12 (16.0)	2 (2.7)	3 (4.0)		
*Klebsiella spp*.	Absent	8 (10.5)	16 (21.1)	20 (26.3)	6 (7.9)	0 (0.0)	0.717	<0.001
	Present	3 (3.9)	7 (9.2)	2 (2.6)	2 (2.6)	2 (2.6)		
*Enterbacter spp*.	Absent	11 (14.7)	22 (29.3)	21 (28.0)	7 (9.3)	11 (14.7)	2.446	0.654
	Present	0 (0.0)	1 (1.7)	1 (1.7)	1 (1.7)	0 (0.0)		
*Pseudomonas spp*.	Absent	11 (14.7)	21 (28.0)	21 (28.0)	7 (9.3)	11 (14.7)	2.595	0.628
	Present	0 (0.0)	2 (2.7)	1 (1.7)	1 (1.7)	0 (0.0)		
*Citro. Spp*.	Absent	10 (13.3)	21 (28.0)	20 (26.7)	7 (9.3)	10 (13.3)	0.109	0.999
	Present	1 (1.3)	2 (2.7)	2 (2.7)	1 (1.3)	1 (1.3)		

**Table 6:** Associations between bacterial organisms and the sheep sex.

Bacterial isolates	Status	Sex		*X* ^2^	*P*-value
	Female freq (%)	Male freq (%)		
*M. haemolytica*	Absent	21 (28.0)	13 (17.3)	0.005	0.567
	Present	25 (33.3)	3 (4.0)		
*P. multocida*	Absent	32 (42.7)	20 (26.7)	0.003	0.577
	Present	14 (18.7)	9 (12.0)		
*S. aureus*	Absent	22 (29.3)	18 (24.0)	1.450	0.167
	Present	24 (32.0)	11 (14.7)		
*E. coli*	Absent	30 (40.0	17 (22.7)	0.331	0.369
	Present	16 (21.3)	12 (16.0)		
*Klebsiella spp*	Absent	0 (0.0)	24 (31.6)	10.87	0.028
	Present	10 (13.2)	5 (19.7)		
*Enterobacter spp*.	Absent	44 (58.7)	28 (37.3)	0.846	0.669
	Present	2 (2.7)	1 (1.7)		
*Pseudomonas spp*.	Absent	43 (57.3)	28 (37.3)	0.333	0.496
	Present	3 (4.0)	1 (1.3)		
*Citrobacter spp*.	Absent	43 (57.3)	25 (33.3)	1.111	0.255
	Present	3 (4.0)	4 (5.3)		

All the bacteria were isolated from those in poor condition. Enterobacter spp was recorded at 0% in sheep with a good body condition score, whilst *Citrobacter spp* and *Enterobacter spp* were not found in sheep that were obese. *Mannheimia haemolytica* was found in sheep with fair and good body condition scores at an equal rate of 17.3%. *Pasteurella multocida* was mostly isolated from sheep with fair body condition, as presented in Table 7.

**Table 7:** Associations between bacterial organisms and the sheep body condition score.

Bacterial isolates	Status	Body condition score				*X* ^2^	*P*-value
	Poor freq (%)	Fair freq (%)	Good freq (%)	Obese freq (%)		
*M. haemolytica*	Absent	8 (10.7)	13 (17.3)	8 (10.7)	5 (6.7)	0.878	0.831
	Present	8 (10.7)	13 (17.3)	13 (17.3)	7 (9.3)		
*P. multocida*	Absent	12 (16.0)	16 (21.3)	15 (20.0)	9 (12.0)	1.209	0.751
	Present	4 (5.3)	10 (13.3)	6 (8.0)	3 (4.0)		
*S. aureus*	Absent	10 (13.3	12 (16.0)	12 (16.0)	6 (8.0)	0.128	0.740
	Present	6 (8.0)	14 (8.7)	9 (12.0)	6 (8.0)		
*E. coli*	Absent	12 (16.0)	16 (21.3)	11 (14.7)	8 (10.7)	2.086	0.555
	Present	4 (5.3)	10 (13.3)	10 (13.3)	4 (5.3)		
*Klebsiella spp*.	Absent	11 (14.7)	21 (28.0)	18 (24.0)	10 (13.3)	1.787	0.618
	Present	5 (6.7)	5 (6.7)	3 (4.0)	2 (2.7)		
*Enterobacter spp*.	Absent	14 (18.7)	25 (33.3)	21 (28.0)	12 (16.0)	1.787	0.618
	Present	2 (2.7)	1 (1.3)	0 (0.0)	0 (0.0)		
*Pseudomonas spp*.	Absent	15 (20.0)	26 (34.7)	20 (26.7)	10 (13.3)	4.558	0.207
	Present	1 (1.3)	0 (0.0)	1 (1.3)	2 (2.7)		
*Citrobacter spp*.	Absent	15 (20.0)	23 (30.7)	18 (24.0)	12 (16.0)	1.787	0.618
	Present	1 (1.3)	3 (4.0)	3 (4.0)	0 (0.0)		

### 3.4 Antibiotic susceptibility

Table 8 shows that *M. haemolytica* and *P. multocida* were highly susceptible to cefotaxime (95% and 100%), ciprofloxacin (83% and 91%), and ampicillin (76% and 65%). Conversely, they were resistant to amoxicillin (66% and 78%) and doxycycline (76% and 74%).

**Table 8:** Antimicrobial susceptibility pattern of *M. haemolytica* and *P. multocida* isolates.

Antimicrobial	Disks conc. (μg)	Performance	Spp of bacteria tested	
		*M. haemolytica N* = 41 (%)	*P. multocida N* = 23 (%)
Ampicillin	30	Susceptible	31 (76)	15 (65)
		Resistant	10 (24)	8(35)
Amoxycillin	30	Susceptible	14 (34)	5 (22)
		Resistant	27 (66)	18 (78)
Cefotaxime	5	Susceptible	39 (95)	23 (100)
		Resistant	2 (5)	0 (0)
Doxycycline	30	Susceptible	9 (24)	6 (26)
		Resistant	31 (76)	17 (74)
Penicillin G	10 (IU)	Susceptible	21 (51)	11 (48)
		Resistant	20 (49)	12 (52)
Ciproflaxacin	30	Susceptible	34 (83)	21 (91)
		Resistant	7 (17)	2 (9)
Chloramphenicol	30	Susceptible	16 (39)	13 (57)
		Resistant	25 (61)	10 (43)
Sulphamethoxazole	25	Susceptible	9 (22)	12 (52)

Table 9 indicates that *Staphylococcus aureus* was sensitive to gentamicin (94%), cefoxitin (71%), and ciprofloxacin (63%), but resistant to tetracycline (63%). In contrast, *Pseudomonas spp*. was completely susceptible to gentamicin, meropenem, and amikacin (all 100%) while resistant to tetracycline (100%) and sulfamethoxazole (75%).

**Table 9:** Antimicrobial susceptibility pattern of *Staphylococcus aureus* and *Pseudomonas* spp.

Antimicrobial	Disks conc. (μg)	Performance	Spp of bacteria tested	
		*Staphylococcus aureus N* = 35 (%)	*Pseudomonas* spp *N* = 4 (%)
Ciprofloxacin/	5	Susceptible	22 (63)	3 (75)
		Intermediate	5 (14)	1 (25)
		Resistant	8 (23)	0 (0)
Tetracycline	30	Susceptible	6 (17)	0 (0)
		Intermediate	7 (20)	0 (0)
		Resistant	22 (63)	4 (100)
Gentamicin	10	Susceptible	33 (94)	4 (100)
		Intermediate	1 (3)	0 (0)
		Resistant	1 (3)	0 (0)
Sulphamethoxazole	25	Susceptible	18 (51)	1 (25)
		Intermediate	1 (3)	0 (0)
		Resistant	16 (46)	3 (75)
Cefoxitin	10	Susceptible	25 (71)	–
		Intermediate	3 (9)	–
		Resistant	7 (20)	–
Clindamycin		Susceptible	16 (46)	–
		Intermediate	6 (17)	–
		Resistant	13 (37)	–
Meropenem	10	Susceptible	17 (49)	4 (100)
		Intermediate	6 (17)	0 (0)
		Resistant	12 (34)	0 (0)
Amikacin	30	Susceptible	**–**	4 (100)
		Intermediate	**–**	0 (0)
		Resistant	**–**	0 (0)
Ceftriaxone	30	Susceptible	**–**	2 (50)
		Intermediate	–	2 (50)
		Resistant	**–**	0 (0)

Table 10 reveals that *Klebsiella spp., E. coli*, and *Citrobacter spp*. were all susceptible to ciprofloxacin and gentamicin (93%, 100%, and 100%), ceftriaxone (93%, 96%, and 100%), and amikacin (100% for Klebsiella and Citrobacter and 96% for *E. coli*), but resistant to tetracycline (67%, 62%, and 57%).

**Table 10:** Antimicrobial susceptibility pattern of *Klebsiella spp., E. coli and Citrobacter* spp.

Antimicrobial (μg)	Performance	Species of bacteria tested		
*Klebsiella* spp *N* = 15 (%)	*E. coli N* = 28(%)	*Citrobacter* spp *N* = 7 (%)
Ciprofloxacin (5)	Susceptible	14 (93)	28 (100)	7 (100)
	Intermediate	1 (7)	0 (0)	0 (0)
	Resistant	0 (0)	0 (0)	0 (0)
Tetracycline (30)	Susceptible	5 (33)	9 (32)	3 (43)
	Intermediate	0 (0)	2 (7)	0 (0)
	Resistant	10 (67)	17 (61)	4 (57)
Gentamicin (10)	Susceptible	14 (93)	28 (100)	7 (100)
	Intermediate	0 (0)	0 (0)	0 (0)
	Resistant	1 (7)	0 (0)	0 (0)
Ceftriaxone (30)	Susceptible	14 (93)	27 (96)	7 (100)
	Intermediate	1 (7)	0 (0)	0 (0)
	Resistant	0 (0)	1 (4)	0 (0)
Sulphamethoxazole (25)	Susceptible	8 (53)	15 (54)	3 (43)
	Intermediate	0 (0)	2 (7)	0 (0)
	Resistant	7 (47)	11 (39)	4 (43)
Meropenem (10)	Susceptible	11 (73)	20 (71)	7 (100)
	Intermediate	1 (7)	0 (0)	0 (0)
	Resistant	3 (20)	8 (29)	0 (0)
Amikacin (30)	Susceptible	15 (100)	27 (96)	7 (100)
	Intermediate	0 (0)	1 (4)	0 (0)
	Resistant	0 (0)	0 (0)	0 (0)

## 4 Discussions

This investigation identified eight bacterial species from pneumonic lung tissues, with *M. haemolytica* (54.7%) and *P. multocida* (30.7%) as the predominant *Pasteurella spp*. Similar studies identified them as the principal causative agents of ovine pneumonic pasteurellosis (15, 16). The high incidence of *M. haemolytica* isolation is associated with its pathogenicity, as it generates leukotoxins and endotoxins capable of inducing fibrinous and necrotising pneumonia (17). The presence of *P. multocida* (30.7%) shows that it is involved in chronic or subacute pneumonia. *Pasteurella multocida* is found to be a prominent cause of bacterial pneumonia in sheep (18).

Bacteria such as *Staphylococcus aureus* (46.7%), *E. coli* (37.3%), and *Klebsiella spp*. (19.7%), were also found. These are characterized as secondary invaders that exacerbate disease severity when host defenses are impaired (19). *Mannheimia. haemolytica, P. multocida, S. aureus, E. coli*, and *Klebsiella pneumoniae* were identified in the lungs of pneumonic sheep in Nigeria, indicating the importance of both primary and secondary bacterial infections (18). The high rate of *S. aureus* isolation in this study agrees with the similar findings (20), and similar results for *E. coli* and *Klebsiella spp*. have been reported (21, 22). This shows how complicated ovine pneumonia is.

*Mannheimia haemolytica* and *Pasteurella multocida* are well-established primary respiratory pathogens in sheep, commonly associated with pneumonic pasteurellosis and significant economic losses in small ruminants. In contrast, *E. coli, Citrobacter spp.*, and *Enterobacter spp*. are generally regarded as opportunistic or environmental organisms, often associated with gastrointestinal or urinary tract infections and septicemia rather than as primary agents of ovine pneumonia.

In addition to the expected *Pasteurella spp* we isolated *Citrobacter spp*. and *Enterobacter spp*. from the lungs of pneumonic sheep. These organisms are rarely reported as primary causes of ovine pneumonia and are more commonly recognized as opportunistic enteric or environmental bacteria in ruminants. This suggests a potentially emerging role for non-classical respiratory pathogens in ovine pneumonia. These findings hold epidemiological significance as they indicate possible shifts in the causative agents of pneumonia in small ruminants. They also underscore the need to consider such opportunistic Enterobacteriaceae when developing empirical treatment protocols and AMR surveillance frameworks for West African sheep flocks.

In this study, *M. haemolytica* was the most frequently isolated bacterium from lungs showing broncho-interstitial pneumonia (29.3%) and interstitial pneumonia (17.3%) on histopathology. This pattern is consistent with previous reports that link *M. haemolytica* to fibrinous and interstitial pulmonary lesions in sheep, and suggests an important role for this organism in the pathology of ovine pneumonia in our setting (23). *P. multocida* was also found, especially in cases of interstitial pneumonia (13.3%) and broncho-interstitial pneumonia (9.3%). This shows that it is involved in both chronic and acute respiratory disorders (24). *M. haemolytica* and *P. multocida* each made up 8.0% of bronchopneumonia cases, and this is comparable to the findings of Tuzcu et al. (25). *S. aureus* and *E. coli* were also found in all kinds of pneumonia. They may be opportunistic pathogens and are often seen as secondary invaders (26).

The study found that *M. haemolytica* (30%) and *P. multocida* (16%) were more prevalent during the dry season. This shows that dry weather and stress make respiratory disorders more common in small ruminants (15). Furthermore, the high incidence of *P. multocida* in the dry season was identified in pneumonic pasteurellosis during periods of environmental stress (21).

*M. haemolytica* was the main pathogen found in all breeds, showing that it is very important for ovine pneumonia. Sahelian breeds had all types of bacteria, which means they may be more susceptible to pneumonia. The West African Dwarf sheep had most of the isolates, except for *Enterobacter spp*. This shows that both breed types are at risk for respiratory diseases.

The presence of *M. haemolytica, P. multocida, S. aureus*, and *E. coli* in sheep aged 2 to 3 years is comparable to a similar study where *Pasteurella spp* were more often found in younger to middle-aged sheep, but less often in older ones (27). This may be due to young adults being more exposed to bacterial pneumonia than lambs or older animals.

Higher female isolates may relate to prolonged flock exposure, warranting farm-level studies (24). This management-related characteristic rather than an inherent sex-linked susceptibility.

The heightened recovery of pathogens from sheep in fair and bad body condition may be indicative of the importance of nutritional and managerial stress in pneumonia. Animals are more likely to get bacterial infections if they are sick (28). Sheep in poor body condition present more pronounced lung lesions and elevated bacterial isolation rates (24).

In this study, *M. haemolytica* and *P. multocida* were sensitive to cefotaxime (95% and 100%), ciprofloxacin (83% and 91%), and ampicillin (76% and 65%), respectively. These findings suggest that fluoroquinolones and third-generation cephalosporins continue to be effective therapies for ovine pneumonia in the examined area. Reports have demonstrated similar patterns of susceptibility, with *M. haemolytica* and *P. multocida* being quite sensitive to ciprofloxacin and cefotaxime (16, 29). Other investigations showed that cephalosporins and fluoroquinolones worked well against Pasteurella species taken from small ruminants (21, 30).

Some studies have revealed contradictory results concerning *P. multocida*. A study in the northern part of Tanzania reported that *P. multocida* was susceptible to gentamicin and ciprofloxacin but exhibited considerable resistance to cefotaxime and other β-lactams (31). Similarly, another study found that 75% of *P. multocida* isolates were resistant to cefotaxime (32). These disparities may arise from differences in the dissemination of resistance genes, drug availability, and regional trends in antimicrobial utilization.

This study contradicts a finding that showed tetracyclines were effective against respiratory infections in small ruminants in Tanzania and Uganda, respectively (1). Tetracycline resistance was high in Ethiopia, Nigeria, and Ghana, underscoring the imperative for judicious application of this antibiotic to preserve its effectiveness (33).

*Mannheimia. haemolytica* and *P. multocida* showed resistance to amoxicillin, with resistance rates of 66%, and 78%, respectively. β-lactam antibiotic resistance in *Pasteurella spp*. *M. haemolytica* also showed resistance to chloramphenicol (78%) and sulfamethoxazole (61%) (34). The high levels of resistance are probably because these antibiotics are used a lot and not always in a controlled way in smallholder livestock systems. Additionally, previously effective broad-spectrum antibiotics are losing their effectiveness. *P. multocida* showed a high level of resistance to penicillin G (52%), which supports what Peng et al. (35) found. This trend underscores the escalating issue of antimicrobial resistance (AMR) in bacterial infections of small ruminants, attributable to the misuse of easily accessible drugs such as sulphonamides, amoxicillin, and penicillin (36).

The present study agreed with the finding revealing that *Staphylococcus aureus* isolates exhibited considerable resistance to tetracycline (63%) while showing high susceptibility to gentamicin (94%), cefoxitin (71%), and ciprofloxacin (63%) (34). However, a study in Tanzania found that *S. aureus* was less sensitive to ciprofloxacin than to β-lactams and aminoglycosides (37). The present investigation indicates that aminoglycosides, especially gentamicin, continue to be viable treatment alternatives. On the other hand, the resistance to tetracycline that was seen shows how difficult it might be to utilize this antibiotic for a long time in sheep production.

In this study, all *Pseudomonas spp*. isolates exhibited 100% sensitivity to gentamicin, meropenem, and amikacin, significantly surpassing recent findings. A susceptibility rate of just 76.8% to amikacin and 52% to meropenem, raising concerns regarding the diminished efficacy of gentamicin (38). A study in Northern Iran proposed limiting amikacin use to urine isolates due to differing susceptibility (39). Resistance trends are on the rise, with gentamicin resistance reaching 28.8% (40) and meropenem resistance ranging from 23 to 50% in different areas (41).

Gentamicin and amikacin are often effective against *Pseudomonas spp*, with meropenem demonstrating 100% susceptibility in severe infections, despite the emergence of carbapenem-resistant bacteria (42).

*Klebsiella spp., E. coli*, and *Citrobacter spp*. from sheep with pneumonia were identified and found to be very sensitive to ciprofloxacin, gentamicin, ceftriaxone, and amikacin (93% to 100%), but resistant to tetracycline (57% to 67%). These findings are consistent with previous studies that validate the effectiveness of ciprofloxacin and gentamicin against these infections (43). This study found susceptibility rates of ceftriaxone in *Klebsiella* spp and *E. coli* varied between 93 and 96%. These rates surpass those noted in other regions where resistance to third-generation cephalosporins is frequently found, attributed to the rise of extended-spectrum beta-lactamase (ESBL)–producing Enterobacteriaceae; this aligns with reports of ESBL-producing E. coli on Ashanti farms, suggesting that similar resistance mechanisms may be present in small-ruminant production systems in our setting (44). It is also well known that *Citrobacter spp* are commonly resistant to many drugs, especially beta-lactams like ceftriaxone. Nonetheless, these species may exhibit sensitivity to aminoglycosides such as gentamicin and amikacin, as demonstrated by a 100% susceptibility rate in this study (45).

The recovery of resistant *S. aureus, E. coli, Klebsiella spp*. and *Pseudomonas spp*. from the lungs of sheep entering the food chain is particularly important because these organisms are also among the leading resistant pathogens in human, animal and food isolates in Ghana. Reports of a resistant *E. coli* suggest that AMR is interconnected in animals and humans. The presence of *E. coli* and other Gram-negative bacteria in food animals in Ghana and the discovery of ESBL-producing strains on farms in the Ashanti Region of Ghana (46). It is important to note that the primary sources of *Enterococcus faecalis* respiratory resistance in Ghana are *Enterococcus faecalis* and *Staphylococcus aureus*, both of which enter the food chain through the lungs of sheep (47). Carcass contamination during slaughter and processing can facilitate the transfer of these bacteria or their resistance genes to meat handlers and consumers (48), adding to the documented burden of multidrug-resistant Enterobacteriaceae and staphylococci in raw meat and other animal-source foods. Our findings, therefore, extend beyond animal health, signaling a potential pathway for foodborne exposure to resistant bacteria and underscoring the need for integrated surveillance and control measures that link on-farm antimicrobial use, abattoir hygiene and public health interventions along the meat value chain.

The antimicrobial susceptibility patterns reported in this study highlight the importance of antimicrobial stewardship in small-ruminant medicine. While several of the tested agents, including [list key drugs, e.g., cefotaxime and ciprofloxacin], showed high *in vitro* activity against the isolated respiratory pathogens, these antimicrobials are classified as critically important for both veterinary and human health. Their use should therefore be restricted to situations where narrower-spectrum or lower-priority options are inadequate or unavailable.

Future studies should undertake larger population-based or longitudinal studies of sheep flocks to verify the documented risk factors and delineate time associations regarding pneumonia incidence. A surveillance of antimicrobial resistance, ideally within a One Health framework, using a combination of culture-independent molecular diagnostics, is what will be required to fully elucidate the pathogen spectrum, resistance mechanisms and impacts on animal and public health in Ghana and the rest of West Africa.

## 5 Limitations of the study

The study is subject to several limitations. Due to its abattoir-based cross-sectional design, the causal links between risk variables and pneumonia cannot be demonstrated, and sheep slaughtered may not represent the larger population. A potential limitation of this study is the risk of misclassification of pneumonia histotypes and bacterial status. Histopathological classification into bronchopneumonia, interstitial, and broncho-interstitial patterns is subject to some degree of observer variability, and culture-based methods may fail to detect fastidious or non-viable organisms. Any such misclassification is likely to be non-differential with respect to abattoir and season, and could have biased some of the observed associations toward the null. Moreover, one should be cautious in interpreting the *p*-values for the sparse tables, because the sample size yielded small cell counts for several comparisons, and we primarily relied on Pearson's chi-square tests. Antimicrobial susceptibility testing did not include all possible quality control measures. Bacteriological identification was based only on culture and biochemical techniques without any confirmation analysis by molecular methods. The study relied only on conventional phenotypic methods for identifying *M. haemolytica* and *P. multocida* at the species level. While common in veterinary labs, these methods lack the specificity of molecular techniques like PCR or sequencing, risking some misclassification within Pasteurellaceae. Future studies should use species-specific PCR and sequencing to validate findings. These factors may limit the accuracy and transparency of some results and were considered when planning the future research and extrapolation of our results.

## 6 Conclusion

*M. haemolytica* was the most prevalent bacterial pathogen isolated from pneumonic sheep lungs, suggesting its primary role alongside *S. aureus* associated with pneumonia in sheep assessed at abattoirs in Ghana. Other bacteria identified are *Pasteurella multocida, E. coli, Klebsiella spp., Pseudomonas spp., Enterobacter*, and *Citrobacter spp*. The prevalence of the disease changes with the seasons, and it affects younger sheep and those in worse body condition more. *Klebsiella spp*. is particularly associated with age. These bacteria are mostly susceptible to antibiotics such as cefotaxime, ciprofloxacin, and gentamicin, but they are not eliminated by amoxicillin, tetracycline, and penicillin G. Farmers should contact a veterinarian early when sheep show signs of pneumonia, follow the full prescribed treatment course with effective drugs rather than stopping treatment once clinical signs improve.

## Data Availability

The raw data supporting the conclusions of this article will be made available by the authors, without undue reservation.
